# Artificial intelligence algorithms based approach in evaluating COVID-19 patients and management

**DOI:** 10.2478/jccm-2025-0032

**Published:** 2025-07-31

**Authors:** Ioana Hălmaciu, Anca Meda Văsieșiu, Andrei Manea, Andrei Dragomir, Ioana Tripon, Vlad Vunvulea, Cristian Boeriu, Andrea Rus, Minodora Dobreanu

**Affiliations:** Department of Radiology, George Emil Palade University of Medicine, Pharmacy, Science, and Technology of Targu Mures, Targu Mures, Romania; Department of Infectious Diseases, George Emil Palade University of Medicine Pharmacy Science, and Technology of Targu Mures, Targu Mures, Romania; Doctoral School of Medicine and Pharmacy, George Emil Palade University of Medicine, Pharmacy, Science, and Technology of Targu Mures, Targu Mures, Romania; Faculty of Medicine, George Emil Palade University of Medicine, Pharmacy, Science, and Technology of Targu Mures, Targu Mures, Romania; Department of Anatomy, George Emil Palade University of Medicine, Pharmacy, Science, and Technology of Targu Mures, Targu Mures, Romania; Department of Emergency Medicine, George Emil Palade University of Medicine, Pharmacy, Science, and Technology of Targu Mures, Targu Mures, Romania; Department of Radiology, Clinical Emergency County Hospital of Targu Mureș, Targu Mures, Romania; Center for Advanced Medical and Pharmaceutical Research, George Emil Palade University of Medicine, Pharmacy, Science, and Technology of Targu Mures, Targu Mures, Romania; Department of Laboratory Medicine, George Emil Palade University of Medicine, Pharmacy, Science, and Technology of Targu Mures, Targu Mures, Romania

**Keywords:** COVID-19, SARS-CoV-2, interleukin 6, artificial intelligence, inflammatory markers

## Abstract

**Introduction:**

COVID-19 pneumonia manifests with a wide range of clinical symptoms, from minor flu-like signs to multi-organ failure. Chest computed tomography (CT) is the most effective imaging method for assessing the extent of the pulmonary lesions and correlates with disease severity. Increased workloads during the COVID-19 pandemic led to the development of various artificial intelligence tools to enable quicker diagnoses and quantitative evaluations of the lesions.

**Aim of the study:**

This study aims to analyse the correlation between lung lesions identified on CT scans and the biological inflammatory markers assessed, to establish the survival rate among patients.

**Methods:**

This retrospective study included 120 patients diagnosed with moderate to severe COVID-19 pneumonia who were admitted to the intensive care unit and the internal medicine department between September 2020 and October 2021. Each patient underwent a chest CT scan, which was subsequently analysed by two radiologists and an AI post-processing software. On the same day, blood was collected from the patients to determine inflammatory markers. The markers analysed in this study include the neutrophil-lymphocyte ratio (NLR), monocyte-lymphocyte ratio, platelet-lymphocyte ratio, systemic immune-inflammatory index, systemic inflammation response index, systemic inflammation index, and serum interleukin-6 value.

**Results:**

There were strong and very strong correlations between the derived inflammatory markers, interleukin-6, and the CT severity scores obtained by the AI algorithm (r=0.851, p<0.001 in the case of NLR). Higher values of the inflammatory markers and high lung opacity scores correlated with a decreased survival rate. Crazy paving was also associated with an increased risk of mortality (OR=2.89, p=0.006).

**Conclusions:**

AI-based chest CT analysis plays a crucial role in assessing patients with COVID-19 pneumonia. When combined with inflammatory markers, it provides a reliable and objective method for evaluating COVID-19 pneumonia, enhancing the accuracy of diagnosis.

## Introduction

Severe acute respiratory syndrome coronavirus 2 (SARS-CoV-2) infection was declared a global pandemic by the World Health Organisation on 11 March 2020.

The symptomatic course of SARS-CoV-2 infection varies and progresses gradually, starting with mild flu-like symptoms such as fever, dry cough, and fatigue, then advancing to severe pneumonia, acute respiratory distress syndrome, and multiple organ failure, often requiring intensive therapy. At this stage, studies in the literature report a low survival rate among these patients [1,2].

According to World Health Organisation guidelines, the diagnosis of infection with the SARS-CoV-2 virus is conducted using the reverse transcriptase polymerase chain reaction (RT-PCR) test, which remains the gold standard for detecting this disease today [3,4,5].

Because the clinical course can often be unpredictable and misleading in these patients, computed tomography (CT) scanning has been and remains the preferred imaging method for accurately measuring the extent of lung impairment. The degree of lung damage correlates with disease severity and the need for intensive care therapy [6,7].

The main lung lesions reported in the literature among patients with COVID pneumonia were ground-glass opacities (GGO), crazy paving, pulmonary consolidations, interlobular septal thickening and pulmonary nodules with a bilateral diffuse distribution [8,9,10,11].

During the pandemic, the number of chest CT scan examinations increased significantly, making the use of artificial intelligence in medical imaging software an ideal solution for the precise assessment of lung lesions in a shorter timeframe. This helps limit pulmonary complications, establish the appropriate therapeutic protocol, and also decreases the workflow in the radiology department.

In this context, the aim of this study was to analyse whether the assessment of lung lesions’ degree using AI software, in correlation with inflammatory markers extracted from the laboratory data of COVID-19 pneumonia patients included in the study, can provide prognostic information regarding the deterioration of patients’ clinical condition and the risk of mortality.

The AI software used in this study is from Siemens Healthineers, enabling automated lung segmentation and volumetric measurement of both lung parenchyma density and lung lesions. After the analysis, the software produces a final report that includes an accurate severity score for each lung lobe.

This software can also be used for quantifying lung injury in other types of viral pneumonias, such as those caused by viruses other than COVID-19.

## Methods

The study included 168 patients hospitalised between September 2020 and October 2021 at the Emergency Clinical County Hospital of Târgu Mureș, either in the Intensive Care Unit or in the Internal Medicine Department. All patients were confirmed to have COVID-19 infection by RT-PCR test.

The patients underwent a lung CT scan using either a 128 detector row CT (Siemens Somatom Definition AS+) or a 64 detector row CT (Siemens Somatom Definition AS) with a single breath hold. The slices were reconstructed with a sharp lung kernel (B80f) with a slice thickness of 1 mm, and the Digital Imaging and Communications in Medicine (DICOM) images were subsequently processed using a workstation server equipped with Syngo Via VB80A (Siemens Healthcare, Erlangen, Germany).

The criteria on which the patients were selected were:

Inclusion criteria:
  - Patients aged over 18 years.
  - Confirmed positive COVID-19 diagnosis by RT-PCR.
  - DICOM files that met the AI software’s minimum requirements.
  - Patients who underwent a chest CT scan at admission or within a maximum of 48 hours following admission.
  - Complete blood count analysis and IL-6 levels performed on the same day as the chest CT scan.
Exclusion criteria:
  - Patients under the age of 18.
  - Patients who had CT artefacts due to the inability to maintain respiratory apnoea during the examination, which the software could not process.
  - DICOM files that did not meet the minimum requirements of the software.

**Fig. 1.**
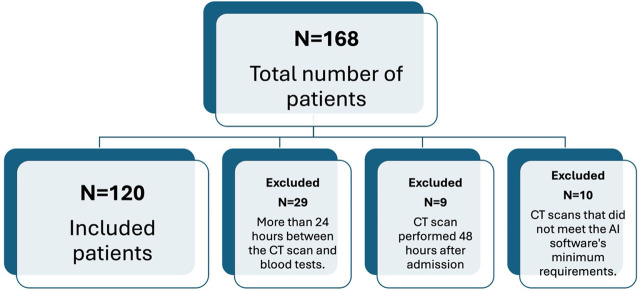
Flow diagram of study participants

After applying the inclusion and exclusion criteria, the number of eligible patients remaining in the study was 120 (Figure 1). Analysis of the CT examinations was performed conventionally by two radiologists and automatically by the AI software. The report produced by the two radiologists included a qualitative, COVID pneumonia-specific assessment of lesions such as GGO, crazy paving, consolidations, and pleural collections.

The AI algorithm used was integrated into the CT Pulmonary Density module, which is part of Siemens’ syngo. CT Extended Functionality with software version VB80A (Siemens Healthcare, Erlangen, Germany). The software is part of the George Emil Palade University of Medicine, Pharmacy, Science and Technology of Târgu Mureș Radiology laboratory.

The algorithm’s output consists of a table that includes the total lung volume, the volume of pulmonary lesions graded as opacities and high opacities (≥ −200 HU), presented as both absolute values and percentage, as well as the mean Hounsfield Units (HU), and the standard deviation (SD) of these lesions (opacities and high opacities). Additionally, it provides the opacity score for each lobe and the total opacity score. The scoring system employs five categories (ranging from 0 to 4), based on the ratio of opacity volume to lung lobe volume. These values are summed to yield a score from 0 to 20. This method is based on the scoring approach published by Bernheim et al. in 2020 [12].

**Fig. 2.**
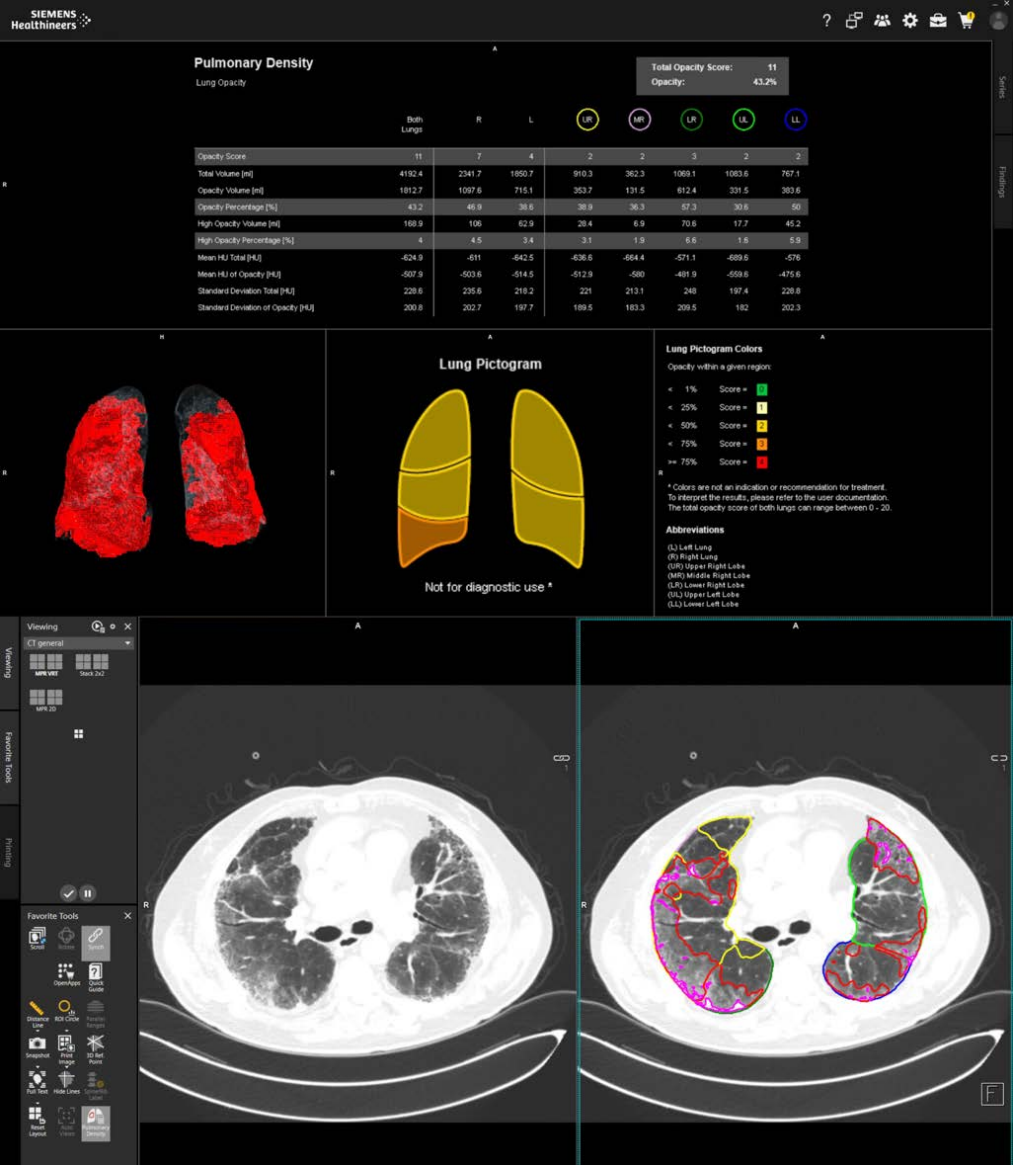
Output of the Siemens CT Pulmonary Density module

Moreover, the algorithm generates a new series of images stored in the PACS system, including an overlay of lung lesions over the original images, a 3D reconstruction of the lung lesions’ locations, and a lung pictogram with the visual scores. This series of images helps radiologists to achieve a faster and more comprehensive diagnosis. The output is demonstrated in Figure 2.

Image evaluation was conducted by two radiologists: M. A., a resident interested in thoracic imaging, and H.I., a senior doctor with over 10 years of experience.

The blood tests included in the study consisted of a complete blood count, measuring haemoglobin, platelets, neutrophils, lymphocytes, monocytes, and interleukin-6 (IL-6) levels. These tests were performed on the same day as the chest CT scan and reflected the patients’ biological response to the infection.

Based on the blood count, we also calculated the neutrophil-to-lymphocyte ratio (NLR), monocyte-to-lymphocyte ratio (MLR), platelet-to-lymphocyte ratio (PLR), systemic immune-inflammation index (SII), systemic inflammation response Index (SIRI), and aggregate index of systemic inflammation (AISI) [13,14].

All data were analysed using IBM SPSS version 22 (IBM Corp, Armonk, NY). The statistical analysis included descriptive and inferential statistical methods, such as normality tests (Shapiro-Wilk), tests for comparing the central tendency (T-Student or Mann-Whitney U tests), correlation tests (Spearman’s rank correlation), and tests for categorical variables (Fisher and Chi-Square tests). A p-value of <0.05 was considered statistically significant, with a 95% confidence interval.

The study was approved by the Ethics Committee of the Emergency County Hospital of Mureș, with the number 10488/29.04.2022. This study is part of the research grant no. 164/16/10.01.2023, with the title “Correlations between inflammatory and nutritional status and the degree of lung damage using artificial intelligence imaging software, among patients with viral pneumonia due to SARS-CoV-2 virus infection, versus other types of viral pneumonia” being financed by the George Emil Palade University of Medicine, Pharmacy, Science and Technology of Târgu Mureș.

## Results

The patient group consisted of 61 (50.8%) male patients and 59 (49.2%) female patients, who were hospitalised between September 2020 and October 2021. All demographic data, blood test results, radiological qualitative assessments, and AI algorithm quantitative evaluations are detailed in Table 1.

**Table 1.** Patient characteristics

**Variable**	**n (%)/ Mean ± SD / Median (IQR)**
**Sex**	
Male	61 (50.8%)
Female	59 (49.2%)

**Qualitative features**	
Crazy paving	57 (47.5%)
GGO	60 (50%)
Pleural effusion	10 (8.3%)
Consolidation	56 (46.7%)

**Discharge status**	
Alive	54 (45%)
Dead	66 (55%)

Age	71.5 (14)

**Blood tests**	
Platelet count	257.25 ± 121.67
Haemoglobin	11.75 ± 2.70
Haematocrit	36.11 ± 7.67
Leukocyte count	10.70 (7.24)
Neutrophil count	9.39 (8.15)
Lymphocyte count	0.81 (0.77)
Monocyte count	0.63 (0.56)
Basophil count	0.02 (0.02)
IL-6	29.16 (65.23)
NLR	2936.39 (3852.17)
MLR	6.30 (8.90)
PLR	10.21 (15.09)
SII	0.65 (0.73)
SIRI	281.41 (307.84)
AISI	1536.77 (2933.83)

**Quantitative Analysis**	
Opacity score	12.00 (11)
Right Lung	5.00 (4)
Left Lung	2.00 (2)
Upper Right Lobe	2.00 (2)
Middle Right Lobe	3.00 (3)
Lower Right Lobe	2.00 (2)
Upper Left Lobe	3.00 (3)
Lower Left Lobe	12.00 (11)
Both Lungs Opacity Volume	1474.95 (1902.90)
Both Lungs Opacity percentage	48.05 (56.70)
Both Lungs High opacity volume	320.25 (514.70)
Both Lungs High opacity percentage	8.50 (16.08)
Both Lungs Mean HU total	−603.00 ± 133.01
Both Lungs Mean HU of Opacity	−461.12 ± 115.27

n - number of patients; SD - standard deviation; IQR – interquartile range. Mean ± SD was used if the values passed the normality test. Median (IQR) was used if the values did not pass the normality test.

We subdivided the patient lot by their state at discharge into two groups: alive and deceased. We compared the mean values of the numeric variables using Student t-tests (for Gaussian distributions) and Mann-Whitney tests (for non-Gaussian distributions), depending on the distribution of the data. We presented the results in Table 2.

**Table 2.** Comparison of the numeric values of the patients

**Variable**	**Discharged alive** **n=54**	**Discharged deceased** **n=66**	**p**
Age	66.07 ± 11.70	72.50 (12.25)	0.024
Platelet count	257.45 ± 121.05	257.09 ± 123.11	0.987
Haemoglobin	11.37 ± 2.48	12.06 ± 2.85	0.161
Haematocrit	35.18 ± 7.02	36.87 ± 8.13	0.231
Leukocyte count	8.38 (6.18)	13.33 (6.96)	<0.001
Neutrophil count	6.43 (7.22)	11.22 (7.57)	<0.001
Lymphocyte count	1.07 (1.05)	0.71 (0.67)	0.022
Monocyte count	0.60 (0.48)	0.70 (0.65)	0.549
Basophil count	0.02 (0.02)	0.02 (0.02)	0.732
IL-6	16.35 (28.53)	56.33 (101.86)	<0.001
NLR	1755.97 (3289.87)	3636.35 (3931.26)	0.001
MLR	3.75 (6.09)	9.07 (12.90)	<0.001
PLR	6.50 (9.30)	12.78 (15.60)	<0.001
SII	0.57 (0.54)	0.75 (0.85)	0.013
SIRI	228.31 (247.29)	347.83 (340.16)	0.038
AISI	1025.25 (1870.20)	2059.01 (3985.77)	0.005
Opacity score	7.00 (10.50)	14.00 (7.00)	<0.001
Right Lung	4.00 (7.25)	9.00 (4.50)	<0.001
Left Lung	2.00 (4.25)	6.00 (3.00)	<0.001
Upper Right Lobe	1.00 (3.00)	3.00 (2.00)	<0.001
Middle Right Lobe	1.00 (2.25)	2.00 (2.00)	<0.001
Lower Right Lobe	2.00 (2.00)	3.00 (1.25)	<0.001
Upper Left Lobe	1.00 (2.25)	2.00 (1.25)	<0.001
Lower Left Lobe	1.00 (2.00)	3.00 (1.00)	0.555
Both Lungs Opacity Volume	605.85 (1779.78)	1850.88 ± 955.79	<0.001
Both Lungs Opacity percentage	18.15 (52.15)	57.55 (30.38)	<0.001
Both Lungs High opacity volume	104.80 (426.18)	383.00 (362.35)	<0.001
Both Lungs High opacity percentage	2.35 (12.43)	12.15 (14.80)	<0.001
Both Lungs Mean HU total	−692.40 (198.05)	−550.74 ± 117.19	<0.001
Both Lungs Mean HU of Opacity	−491.33 ± 124.97	−436.41 ± 101.06	.009

n - number of patients. Mean ± SD was used if the values passed the normality test. Median (IQR) was used if the values did not pass the normality test.

There were statistically significant differences between the two subgroups of patients regarding age; patients discharged deceased had a greater age.

There was also a statistically significant difference in the counts of leukocytes, neutrophils, and lymphocytes; patients who were discharged dead had higher leukocyte and neutrophil values, but lower lymphocyte values.

IL-6 values were also higher in the deceased discharge group, as well as all the inflammatory markers NLR, MLR, PLR, SII, SIRI, and AISI derived from the blood count.

Regarding the chest CT scan results, patients in the discharged alive group tended to have lower opacity score, as well as lower Mean Hounsfield units (HU) of the opacity volume. All the lobe scores had higher values in deceased patients, with the exception that the lower left lobe scores were not significantly different in the two groups.

We used the Chi-Square Test and Fisher’s Exact Test to evaluate associations between sex and the qualitative radiological features of patients with discharged status. In the case of crazy paving, there was an approximately 3 times higher chance (OR=2.89) of death if the patient presented these types of changes, this correlation being statistically significant (p=0.006). For the other qualitative features, there were no statistically significant correlations with the patient’s outcome. The contingency tables used and the p-values are presented in Table 3.

**Table 3.** Categorical variables of the patients

**Variable**	**Discharged alive** **n=54**	**Discharged deceased** **n=66**	**p / OR**
**Sex**			
Male	24 (20%)	37 (31%)	0.205
Female	30 (25%)	29 (24%)	

**Crazy paving**			
No	36 (30%)	27 (23%)	0.006/OR=2.89
Yes	18 (15%)	39 (33%)	

**Ground Glass-Opacities**			
No	27 (23%)	33 (28%)	1
Yes	27 (23%)	33 (28%)	

**Pleural Effusion**			
No	50 (42%)	60 (50%)	1
Yes	4 (3%)	6 (5%)	

n- number of patients; OR – odds ratio

We applied the Spearman correlation test comparing inflammatory markers IL-6, SII, SIRI, NLR, MLR, PLR and AISI with four quantitative radiological features (Both lungs opacity score and percentage, both lungs high opacity percentage, and Both Lungs Mean HU of opacity). Our results indicated that: IL-6 correlates directly with all the radiological features, the corelation with both lungs opacity (BLO) and BLO percentage being strong, statistically significant correlations and the correlation with both lungs high opacity (BLHO) and both lungs (BL) mean HU of opacity being very strong, statistically significant ones. SII, SIRI, NLR, MLR and AISI also show a strong statistically significant correlation with the BLO score and BLO percentage. Only IL-6 and SII correlate statistically significantly with the Mean HU of the opacity of both lungs. All the results from the Spearman test are presented in Table 4.

**Table 4.** Correlation between inflammatory markers and quantitative radiological features

**Inflammatory biomarkers**	**Quantitative radiological features**							

	**BLO score**		**BLO percentage**		**BLHO percentage**		**Both Lungs Mean HU of Opacity**	

	**r**	**p**	**r**	**p**	**r**	**p**	**r**	**p**
IL-6	0.663	0.010	0.682	0.007	0.851	0.000	0.856	0.000
SII	0.844	0.000	0.814	0.000	0.709	0.005	0.626	0.017
SIRI	0.793	0.000	0.812	0.000	0.563	0.036	0.503	0.067
NLR	0.851	<0.001	0.814	<0.001	0.695	0.006	0.503	0.067
MLR	0.742	0.002	0.741	0.002	0.461	0.097	0.371	0.191
PLR	0.691	0.006	0.647	0.012	0.545	0.044	0.429	0.126
AISI	0.766	0.001	0.770	0.001	0.519	0.057	0.516	0.059

*r - r-values, p- p-values; r values* - Spearman correlation coefficients.; *BLO* -both lungs opacity; *BLHO* – both lungs high opacity

We compared the values of the inflammatory markers with the crazy paving qualitative radiological feature. There is a statistically significant difference in the IL-6 values, patients with crazy paving patterns having higher values. There is no significant difference between the values of the other inflammatory markers. All the results are presented in Table 5. Comparing the same markers regarding patient outcome (dead or alive), there was no difference between the groups that had crazy paving and those that did not.

**Table 5.** Inflammatory markers comparison based on the crazy paving pattern

**Inflammatory markers**	**Crazy paving – No** **n=63**	**Crazy paving – Yes** **n=57**	**p- value**
IL-6	10.74 (38.67)	193.24 ± 214.37	0.020
SII	2842.3 ± 2530.29	5232.57 ± 3075.4	0.136
SIRI	5.23 ± 4.58	10.81 ± 6.43	0.081
NLR	9.51 ± 8.17	14.97 ± 4.14	0.162
MLR	0.47 ± 0.28	0.69 ± 0.27	0.167
PLR	235.9 ± 153.01	390.6 ± 250.05	0.176
AISI	1619.87 ± 1514.74	3759.81 ± 2871.04	0.095

n- number of patients. Mean ± SD was used if the values passed the normality test. Median (IQR) was used if the values did not pass the normality test.

## Discussions

Results from this study support the hypothesis that the extent of lung damage independently predicts the risk of clinical deterioration or death during hospitalisation among patients with COVID-19 pneumonia.

In recent years, numerous studies have described CT imaging features of COVID-19 pneumonia[8,15,16,17,18]. These are primarily characterised in the early exudative phase, during the first five days, by GGO, arranged peripherally subpleural, bilaterally, affecting several lung lobes, with subsequent progression towards pulmonary consolidation (up to 14 days) through intra-alveolar organisation, fibrotic proliferation, and alveolar collapse [12,19,20].

Pulmonary consolidation marks the peak stage of COVID-19 pneumonia and is characterised by intense inflammatory changes, with extensive alveolar lesions, resembling organised pneumonia. At this stage, patients may enter the critical phase of the disease [8,21,22].

In line with existing studies in the literature, the analysis conducted by the AI software in our study revealed a statistically significant difference in the volumes of pulmonary condensations between the two groups classified as survivors and deceased. In the study, Wang and colleagues applied 2D and 3D deep learning models to explore the use of artificial intelligence in CT image analysis, aiming to detect, quantify, and monitor the progression of COVID-19 in patients [8,23].

Among the volumes analysed in this study, consistent with findings from the specialised literature, the GGO volume was predominant at 50%, with a peripheral distribution across several lobes [8,24,25,26,27]. Statistically significant differences were observed between the two lungs, with the right being more affected. Additionally, statistically significant differences were observed between the groups classified based on the radiological severity score, specifically in terms of the volume of remaining healthy lung parenchyma, the volume of GGO, and the volume of pulmonary consolidations.

Grassi et al. conducted a study to quantify lung damage in patients with COVID-19 pneumonia using different software applications for analysing chest CT scans, aiming to stratify patients based on disease severity. The results support the theory that lung lesion quantification assisted by artificial intelligence software is a simple, feasible method with proven efficiency for classifying COVID-19 cases by severity [28].

Among the lung lesions analysed, the presence of the crazy paving pattern was linked with a low survival rate. The findings are consistent with the literature, as Contreras-Grande et al.’s 2021 study, which involved 254 COVID-19 patients, concluded that crazy paving and a high lung CT severity score were associated with greater clinical severity and mortality. A 2020 study by Chon et al., involving 281 patients, demonstrated that crazy paving patterns are associated with critical events [29,30].

The results of our study are consistent with data from the specialised literature; thus, the radiological severity score, in conjunction with biological parameters, provides additional prognostic value among patients with COVID-19 pneumonia.

After discharge, thoracic CT scans are necessary for monitoring fibrotic changes of the lung parenchyma. A study by Stoian et al. from 2023 found mild and severe fibrosis six months after discharge following COVID-19 infection, but it did not correlate with the duration of mechanical ventilation [31].

Among the biological variables analysed, we recorded statistically significant differences between the two groups, survivors versus non-survivors (deceased), for all inflammatory markers analysed: number of leukocytes, neutrophils, lymphocytes, IL-6, NLR, MLR, and PLR ratio, respectively, as well as SII, SIRI, and AISI.

Of these, IL-6 and SII, respectively, were strongly correlated with the presence of pulmonary condensations, which represent the peak stage of COVID-19 pneumonia. In conclusion, IL-6 and SII can be considered predictors for unfavourable clinical evolution among these patients.

Consistent with our study’s findings, Lassau et al. showed that higher neutrophil count and neutrophil-to-lymphocyte ratio are linked to a poorer prognosis in patients with COVID-19 pneumonia [32].

Studies in the literature indicate that serum IL-6 levels positively correlate with the extent of lung damage, as measured on chest CT scans, making it an ideal marker for monitoring the disease. It is the most sensitive among the inflammatory markers studied [24,33,34,35].

Following statistical analysis, our study’s results indicate that the NLR, MLR, PLR, SII, SIRI, and AISI ratios positively correlate with the lung severity score, percentage of opacities, percentage of high opacities, and mean HU values of lesions. The results are in agreement with findings in the literature. Man et al. demonstrated that NLR, along with PLR and eosinophils, positively correlated with an increased lung damage score [36].

Our results suggested that a quantitative assessment of lung injury, aided by artificial intelligence software, can function as an independent prognostic marker for patients with COVID-19 pneumonia. Imaging parameters aligned with the analysed biological markers are independent factors for clinical risk stratification among these patients.

Compared to the quantitative visual assessment of lung lesions performed by radiologists, the use of artificial intelligence software offers greater efficiency, high reproducibility, reduces analysis time, and provides prognostic scores for disease severity with improved predictability, unlike the quantitative visual scores issued by radiologists [37].

AI technologies are not created to replace healthcare professionals; they aim to enhance diagnostic precision and the efficiency of patient care. In the future, radiologists will not be supplanted by AI, but rather by radiologists who adopt AI software in their daily work [37].

### Limitations

The study is retrospective and includes a relatively small number of patients from a single hospital.

## Conclusion

The inflammatory markers analysed in this study were positively correlated with the lung injury score, which indicates that they can be utilised in the assessment and stratification of disease severity in patients with COVID-19 pneumonia.

Validation of these results through a future study, currently in the preliminary phase, will be expanded to include groups of patients with various types of viral pneumonia. This study aims to correlate imaging changes evaluated with AI software with inflammatory biomarkers, with the goal of establishing a prognostic score based on AI evaluation and inflammatory markers to stratify disease severity among these patients.
